# Investigating male factors and their relationships with reproductive health outcomes: a case-control study protocol for Towards Optimal Fertility, Fathering, and Fatherhood studY (TOFFFY) in Singapore

**DOI:** 10.1136/bmjopen-2024-088143

**Published:** 2025-01-15

**Authors:** Chee Wai Ku, Jun Wei Pek, Yin Bun Cheung, Melissa D/O Tharmalingam Durgahshree, Melinda Chan, Yie Hou Lee, Keith Godfrey, Fabian Yap, Jerry Kok Yen Chan, See Ling Loy

**Affiliations:** 1 Department of Reproductive Medicine, KK Women's and Children's Hospital, Singapore; 2 Duke-NUS Medical School, Singapore; 3 Temasek Life Sciences Laboratory, Singapore; 4 Department of Biological Sciences, National University of Singapore, Singapore; 5 Program in Health Services and Systems Research and Centre for Quantitative Medicine, Duke-NUS Medical School, Singapore; 6 Tampere Centre for Child, Adolescent and Maternal Health Research, University of Tampere and Tampere University Hospital, Tampere, Finland; 7 Singapore-MIT Alliance for Research and Technology, Singapore; 8 MRC Lifecourse Epidemiology Unit, University of Southampton and Southampton University Hospitals NHS Trust, Southampton, UK; 9 University of Southampton and University Hospital Southampton National Health Service Foundation Trust, National Institute for Health Research Southampton Biomedical Research Centre, Southampton, UK; 10 Department of Paediatrics, KK Women's and Children's Hospital, Singapore; 11 Lee Kong Chian School of Medicine, Nanyang Technological University, Singapore

**Keywords:** Subfertility, Male infertility, Pregnancy

## Abstract

**ABSTRACT:**

**Introduction:**

Despite the global prevalence of low fertility rates, male contributions to fertility and reproductive health outcomes have been understudied. This study aims to investigate the male contribution to fertility and explore the underlying biological mechanisms. Specifically, we aim to (1) identify male factors associated with successful pregnancy, (2) develop a fertility index incorporating modifiable factors for both males and females to predict pregnancy rate and (3) explore the relationship of male modifiable factors with semen parameters and molecular characteristics.

**Methods and analysis:**

We will conduct an unmatched case-control study involving 240 couples with impaired male fertility (cases) and 240 couples with normal male fertility (controls). Between July 2024 and June 2026, we will recruit 480 eligible couples from KK Women’s and Children’s Hospital, Singapore. Male and female participants will complete questionnaires on sociodemographics, general health and lifestyle factors, and their anthropometry and body fat composition will be measured. Blood and semen samples from the male participants will be collected for biochemical, molecular and semen analyses. Predictive male factors will be identified using the least absolute shrinkage and selection operator method, accounting for female factors. We will construct a logistic regression model incorporating both male and female factors to derive a fertility index, which will be evaluated using cross-validation on subsets of the study population. Multivariable linear regression will be used to explore relationships between male modifiable exposures and semen parameters.

**Ethics and dissemination:**

The study protocol has received approval from the Centralised Institutional Review Board of SingHealth (2024/2120), Singapore. Participants will provide written informed consent. Study results will be disseminated through conferences and peer-reviewed scientific journals.

**Trial registration number:**

NCT06293235.

**STRENGTHS AND LIMITATIONS OF THIS STUDY**

- This unmatched case-control study will comprehensively investigate various factors in males, including biological, clinical, molecular, behavioural and environmental characteristics, to understand their relationships with male reproductive health and outcomes, and provide an important data repository for additional data analyses by the local and international scientific community.
- The study will use automated technology integrated with artificial intelligence algorithms to measure sperm DNA fragmentation and assess biofluid oxidative status.
- The participation of Singapore residents from a diverse, multiracial Asian demographic will provide valuable insights with potential relevance to wider Asian contexts.
- There is a potential for recall bias in self-reported behavioural and lifestyle information, which could affect the predictive accuracy for male fertility status and pregnancy rate.

## Introduction

 Singapore has been grappling with a persistently low total fertility rate of 1.1 since 2018, which dropped below 1.0 in 2023.^1^ Despite the implementation of comprehensive pronatalist policies, this rate remains among the lowest globally, much like many developed countries in the region.^2 3^ Based on the WHO global report, approximately 1-in-6 couples experience challenges in achieving pregnancy.^4^ Subfertility is defined as the failure to achieve conception after 12 months of regular, unprotected sexual intercourse.^5 6^ Given the high prevalence of poor fertility globally, it is crucial to thoroughly examine the factors that impact reproductive health. Historically, fertility research has been primarily focused on women,^7^ but preliminary estimates suggest that males are solely responsible for 20–30% of cases and contribute to 50% overall.^8^ However, our understanding of the causes, impact and consequences of male reproductive health remains inadequate. Emerging reports indicate that men with impaired fertility bear a higher disease burden, with an increased risk of incident diseases such as heart disease and cancer, as well as premature mortality compared with men with normal fertility.^9^ Following the Paternal Origins of Health and Disease (POHaD) concept, as an extension of the Developmental Origin of Health and Disease paradigm, there is increasing evidence suggesting that the father’s health may significantly affect sperm quality and the health of the offspring.^10^,^12^ Consequently, gaining an understanding of the role of male factors in reproductive health is vital for developing effective interventions to address the fertility crisis, informing strategies for male health promotion and management, and potentially leading to improved health of future offspring.

Advanced paternal age has been associated with subfertility, delayed embryo development, increased miscarriage risk and preterm birth, which can be attributable to poor DNA integrity and aberrant DNA methylation in sperm at older age.^13 14^ However, the specific age at which reproductive potential begins to decline remains undefined.^13 15^ Some studies report changes starting around age 40;^16^ others indicate pronounced declines in semen quality by age 50.^13 15^ While certain fertility-influencing factors, such as paternal age, may lie beyond an individual’s control, there are numerous modifiable lifestyle choices and environmental factors that significantly impact male reproductive health and function.^7 17 18^ Studies have shown that high paternal body mass index (BMI), heavy smoking and alcohol dependence increase the likelihood of impaired fertility in males or prolong the time to pregnancy (TTP) for couples.^7 12 19^ These factors have been found to impair sperm count, motility, morphology, DNA integrity and induce oxidative stress.^9 17 19 20^ Furthermore, a study revealed that increased BMI alone did not have a negative influence on semen quality; sperm count and density were only reduced in metabolically unhealthy overweight or obese men, highlighting the role of underlying metabolic function in male fertility.^21^

In terms of diet, high paternal consumption of sugar-sweetened beverages and caffeine has been associated with reduced fecundability.^22 23^ Refined sugars, such as in sweets, and high carbohydrate diets have been linked with reduced sperm concentration and impaired motility;^24^ while caffeine is suggested to negatively impact fertility through DNA defects associated with aneuploidy and DNA breaks.^25^ Low moderate-to-vigorous physical activity and more television watching have been associated with lower total sperm count and concentration.^26^ There is evidence demonstrating an association between higher stress level and poorer semen quality.^27^ Reduced sleep duration has been implicated as a cause of reduced testosterone levels and fecundability.^28 29^ Prolonged sitting and exposure to radiant heat were found to induce testicular heat and oxidative stress, leading to spermatogenesis arrest and sperm DNA damage.^30^ Last but not least, there is increasing interest in endocrine-disrupting chemicals such as bisphenol A and phthalates, which have also been associated with reduced sperm concentration, volume, motility and viability.^31^

Nonetheless, the evidence regarding the relationships of the above-mentioned exposures with human semen parameters, male reproductive function and fecundability obtained from the limited epidemiological studies remains inconclusive and controversial.^7 20^ Many of these studies face challenges in methodology, such as data inaccuracies and reporting bias from maternal reports of paternal factors, relying on BMI for adiposity assessment, fragmented research on individual factors, and were conducted solely in Western or European populations.^7^ Few studies have reported that Asian Americans have decreased success with fertility treatments, including lower pregnancy and live birth rates, which may be attributed to a combination of genetic, environmental and cultural factors.^32^ Moreover, there is a lack of functional or molecular mechanisms explaining the link between these male exposures and fertility. Although semen analysis serves as the fundamental laboratory evaluation of male fertility, the semen parameters based on basic sperm physical characteristics, including motility, density, count, volume and morphology, have been shown to be poor predictors of reproductive outcomes and are not able to reliably discriminate between men with fertility issues.^33^ Increasing evidence suggests that markers of sperm DNA integrity may help differentiate men with impaired fertility from those with normal fertility.^34^ The WHO recommends including sperm DNA fragmentation as an extended parameter to better evaluate male fertility.^35^ Single- and double-stranded DNA breaks in sperm have different reproductive consequences.^36^ Increased reactive oxygen species causes single-stranded DNA breaks in sperm. Sperm DNA fragmentation induced by elevated oxidative stress has been proposed as the link between male obesity and impaired fertility.^14^ Double-stranded DNA breaks in sperm have been linked with suboptimal embryo development, poor implantation rates and recurrent miscarriage in couples without a female factor.^37^,^39^ Together with sperm gene expression and DNA methylation, there may be epigenetic signatures in predicting reproductive outcomes and future offspring health.^20 40 41^

**Figure 1:**
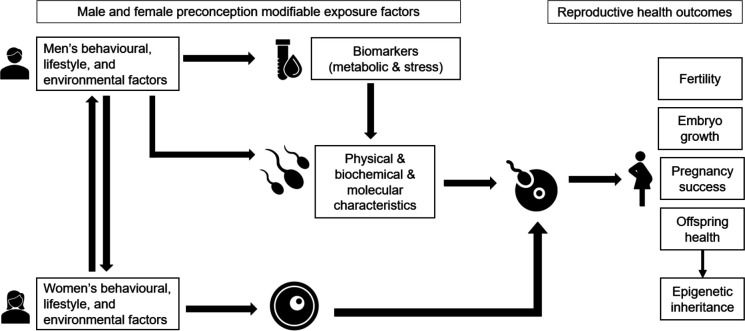
Conceptual framework of the study. The successful outcomes of reproductive health depend not only on female factors but also on male factors, which include behavioural, lifestyle, metabolic and stress exposures, as well as semen parameters. Specifically, sperm molecular characteristics such as oxidative stress, epigenetic signatures including DNA methylation and gene expression may play a role in explaining the connection between male exposures, fertility and the health of offspring.

Therefore, in this study, we will comprehensively examine male factors of fertility, in particular, modifiable exposures, including behavioural, lifestyle and environmental factors, as well as metabolic and stress indicators. For semen parameters, in addition to basic sperm physical characteristics, we will measure sperm DNA fragmentation, DNA methylation and gene expression, as well as seminal plasma oxidative status. Information generated from these data will provide valuable insights regarding their specific relationships with male fertility and reproductive success. Importantly, the study of sperm molecular features has potential implications not only for reproductive outcomes but also for intergenerational effects, predisposing to offspring phenotypes.^20 42^ The conceptual framework of this study is presented in figure 1.

### Objectives

The overall objective of this study is to determine the contribution of male factors to successful pregnancy after accounting for female factors and gain a deeper understanding of the biological mechanisms that underlie this relationship. Specifically, we aim to:

1. Identify male factors associated with the likelihood of pregnancy, independent of female factors.
2. Develop a fertility index that integrates modifiable factors for both males and females to estimate the likelihood of pregnancy.
3. Explore the relationships between male modifiable factors, oxidative status, sperm physical and molecular characteristics.

## Methods and analysis

### Study design and setting

This is an unmatched case-control study involving 240 couples with impaired male fertility (cases) and 240 couples with normal male fertility (controls). Participants are planned to be recruited between July 2024 and June 2026 from KK Women’s and Children’s Hospital (KKH), Singapore. The current protocol is version 1, dated 26 March 2024.

### Participants and recruitment

Couples from the case group will be recruited from the infertility clinics in KKH, while couples from the control group will be recruited from the antenatal clinics in KKH. The team will recruit potential participants through local advertising in the hospital. We will collaborate with obstetricians and gynaecologists at KKH to obtain participant referrals. Moreover, active screening of potential participants will be conducted in the clinics. Other than from the antenatal and infertility clinics, the team will also leverage the Healthy Early Life Moments in Singapore (HELMS) programme to recruit potential cases and controls. HELMS is a new programme established at KKH in April 2022 to provide a continuum care for women with obesity in the general population before, during, and after pregnancy.^43^

### Inclusion criteria

Participants must meet all of the following criteria to be enrolled in this study:

#### Cases

1. Men aged 21–49 years.
2. Men with a female partner aged 21–39 years.
3. Couples who are not able to conceive for at least 12 months of regular, unprotected intercourse.
4. Couples who are Singapore citizens or permanent residents.

#### Controls

1. Men aged 21–49 years.
2. Men with a female partner aged 21–39 years.
3. Men with proven fertility indicated by female partners who are currently pregnant and with viable intrauterine pregnancy at gestational weeks of less than or equal to 16 at the time of the study.
4. Couples with attempted time to conceive within 12 months to achieve this pregnancy.
5. Couples who are Singapore citizens or permanent residents.

### Exclusion criteria

Participants meeting any of the following criteria will be excluded from this study:

#### Cases

1. Male infertility of a known aetiology, including azoospermia, retrograde ejaculation, genetic disorders, cancer treatment, or testicular trauma.
2. Female infertility diagnosis as confirmed by diagnostic imaging or having severe endometriosis.
3. Female partners with an irregular menstrual cycle of >35 days.
4. Couples with known chromosomal abnormalities.

#### Controls

1. Couples who achieve pregnancy after oocyte or spermatozoa donation.
2. Couples with known chromosomal abnormalities.
3. Female partners with known uterine abnormalities.

### Patient and public involvement

In developing the current study protocol, we used feedback from a pilot study of 39 males (Centralised Institutional Review Board 2022/2452) to enhance participant engagement and streamline the study workflow. Feedback regarding willingness to participate, biosample provision and preferred timing of visits informed the planning of our study design and assessments. Additionally, participants in this study will receive their clinical blood and semen test results.

### Data collection and management

During the study visit (the only single timepoint visit), trained research staff will collect data on the sociodemographics and medical history of participants through interviews and medical record collection forms. They will also gather self-reported data on behavioural, lifestyle and environmental factor exposures from the past month using standardised questionnaires. The selection of the recall timeframe is based on the period specified in the validated questionnaires (table 1). Additionally, the staff will conduct anthropometric and body fat composition measurements, and collect blood, urine and semen samples from participants at the clinics in KKH. All data will be saved in a REDCap database. Access to this database is secured with a password, and only approved study team members can access the stored information.

**Table 1:** Data and biosamples collected from both case and control participants

Male partners	Parameters
Sociodemographic, clinical, behavioural, lifestyle and environmental factors	Age, country of origin, length of time in Singapore if born overseas, ethnicity, education, occupation, monthly income, medical history, medication, family history, reproductive health history, male sexual function,^60^ environmental exposure, cigarette smoking, sleep,^61^ stress,^62^ dietary supplement intake, dietary and alcohol intake,^63^ chrononutrition profile,^64^ physical activity,^65^ sedentary behaviour
Anthropometric and blood pressure measures	Body height, weight, body fat composition (InBody 970, USA), blood pressure
Biochemical tests (blood/urine samples)	HbA1c, lipid profile, cortisol, oxidative stress, inflammatory biomarkers, environmental pollutants (endocrine-disrupting chemicals), exosomes, microbial-derived metabolites
Semen analysis	Semen volume, sperm density, sperm motility, sperm morphology, sperm DNA fragmentation
Molecular analysis	mRNA expressions, sncRNA expression, DNA methylation
**Female partners**	
Sociodemographic, clinical, behavioural, lifestyle and environmental factors	Age, country of origin, length of time in Singapore if born overseas, ethnicity, education, occupation, monthly income, medical history, medication, family history, reproductive health history, female sexual function,^66^ environmental exposure, cigarette smoking, sleep,^61^ probable depression,^67^ dietary supplement intake, dietary and alcohol intake,^63^ chrononutrition profile,^64^ physical activity,^65^ sedentary behaviour
Anthropometric and blood pressure measures	Body height, weight and body fat composition (InBody 970, USA), blood pressure

HbA1c, glycated haemoglobin; sncRNA, small non-coding RNA

### Biosampling and biomarker analyses

Male partners of participating couples will be requested to provide peripheral blood, urine and semen samples during their study visit. The blood samples will be processed and analysed for metabolic and stress biomarkers at the biochemical laboratory of KKH within 1–2 hours. The semen samples will be obtained through masturbation after a period of 2–3 days of sexual abstinence. They will be collected in sterile 50 mL non-spermiotoxic polypropylene containers and allowed to liquefy at room temperature. Once the semen has liquefied, basic semen parameters, including volume, density, motility and morphology, will be evaluated in the semen analysis lab of KKH using the LensHooke X12 Pro Semen Analysis System (X12, Bonraybio, Taiwan), following the criteria established by the WHO.^44^ Single- and double-stranded DNA breaks in sperm will be measured using the sperm chromatin dispersion assay (LensHooke R10 Plus kit) and sperm DNA fragmentation releasing assay (LensHooke R11 Plus kit), respectively.^45^ Oxidative status of blood and seminal plasma will be assessed using the point-of-care NMR system.^46 47^ Additional blood and semen samples will be stored at −80°C for future analyses such as inflammatory biomarkers, environmental chemicals and other related biochemical markers.

### Gene expression and DNA methylation analyses

Blood for gene expression analysis will be collected in either Tempus or PAXgene blood RNA tubes, in which total RNA will be extracted. Sperm samples will undergo purification through centrifugation using PureSperm reagents. To extract sperm RNA, we will use an optimised protocol, adapted from Nätt *et al*.^48^ Disulfide bonds will be broken using metal beads and β-mercaptoethanol before RNA extraction using the mirVana RNA isolation kit. Reverse transcription-quantitative real-time PCR will be conducted using the real-time PCR system (Life Technologies, Thermo Fisher Scientific). To determine the fold-change in mRNA expression (normalised against a housekeeping gene GADPH) for a particular participant, we will employ the 2^−ΔΔCt^ method. Additionally, to compare mRNA expression differences between groups, we will use the standard curve method to determine the exact copy amount of RNA.

In addition to mRNAs, we will also characterise the small non-coding RNAs (sncRNAs) and DNA methylation landscapes of human sperm to explore their expression and methylation profiles in relation to paternal modifiable factors and pregnancy status. sncRNAs and DNA methylation have been demonstrated to be regulated by paternal exposures and were suggested to be associated with sperm functionality and embryo development.^4049^,^51^ Genome-wide bisulfite sequencing (to profile global DNA methylation) for sperm from cases and controls will be performed as previously described.^49^ Briefly, genomic DNA from sperm cells will be extracted and treated with bisulfite using the EZ DNA Methylation Lightening Kit (Zymo Research) in accordance with the manufacturer’s protocol. The samples were further subjected to PCR amplification and sequencing using the Illumina platform. Top differentially methylated genes will be selected based on their known functions in spermatogenesis, fertility and metabolic pathways such as insulin signalling, and further verified by targeted bisulfite sequencing using the remaining sperm samples from the cohort.

### Statistical analysis plan

#### Aim 1

We will use multivariable logistic regression model to assess the association between case versus control status and male factors, adjusting for potential confounders such as male sociodemographics, general health, as well as female factors which are correlated with both male factors and successful pregnancy. We will identify these potential confounders through a literature review and conceptual framework, guided by directed acyclic graphs. We will explore propensity score methods, such as inverse probability weighting, to ensure balanced distribution of confounders across case and control groups. We will use the least absolute shrinkage and selection operator regression to select male factors for inclusion in the final model.

#### Aim 2

We will include the identified male factors from aim 1, along with female factors which are known to be associated with successful pregnancy in a multivariable logistic regression. The male factors will focus on those easily accessible measurements. The female factors include age, BMI, smoking exposure, dietary habit, alcohol consumption and folic acid supplementation, which we have identified in our previous study.^52^ We will also include some important clinical data and other maternal factors such as the duration of active conception attempts, sexual health issues and probable depression in the logistic regression to further select the predictors. To ensure accuracy, we will use weighted logistic regression, adjusting the model based on the updated prevalence of successful pregnancies within a year in the population to correct for sampling biases. The best-fitting model will be determined using Akaike’s information criteria. Subsequently, we will create a point-based scoring system based on the final model coefficients,^53^ making it easily applicable in community settings. Integer points will be assigned depending on the presence or absence of each factor, allowing the estimation of overall probability by summing the points. Higher point values will indicate higher chances of pregnancy. To facilitate calculation of the fertility index score, all continuous variables will be categorised based on clinical thresholds or appropriate percentiles prior to the analysis. We will repeat the same procedures by including male metabolic biomarkers and semen parameters that are associated with successful pregnancy in the weighted logistic regression. This will enable the generation of various versions of the fertility index, catering to different levels of complexity for application in primary and tertiary care settings.

Subsequently, we will use 10-fold cross-validation to assess the performance of the derived logistic regression models and respective fertility index scores. This cross-validation procedure helps evaluate the model’s performance on different subsets of the data, providing an estimate of its predictive ability and generalisation performance. The fertility index will be evaluated for its discriminatory ability in predicting chances of pregnancy based on the area under the receiver operating characteristic curve (AUROC), with value >0.80 indicating that the model exhibits a good discriminatory ability or predictive value.

Given that the fertility index comprises male and female factors, we will perform additional analyses to evaluate the individual performance of the index that includes only male factors or female factors using the AUROC. We will also evaluate the performance of the male factor index, with adjustment of female factor index.

#### Aim 3

We will use multivariable linear regression to examine the association between modifiable factors and semen parameters, adjusting for sociodemographic factors.

To understand the relationships between male factors and fertility indices across different conception methods, we will replicate the above analyses by examining outcomes based on assisted and unassisted conception. To assess potential selection bias in both studies, we will compare baseline characteristics between participants with and without a complete set of data. We will use multiple imputation by chained equation to handle missing values.^54^ Sensitivity analyses will be conducted using only samples with complete data. To further ensure robustness, we will test interactions between male and female factors and stratify analyses by key confounders. Statistical analyses will be conducted using the Stata Statistical Software.

### Sample size

There is no straightforward, universally accepted method to estimate the required number of participants for developing a multivariable prediction model. Thus, we employed a few alternative methods to estimate the sample size required. We used paternal BMI as an indicator reflective of paternal lifestyle and metabolic factors that are associated with successful pregnancy and expect that it may be retained in the prediction model. Based on the assumption that males with impaired fertility will have an average BMI 1.3 unit higher than the fertile males,^55 56^ and considering a BMI SD of 4 kg/m^2^, a two-sided 5% type 1 error rate and a power of 90%, the minimum required sample size to detect a difference in BMI between cases and controls is 400 couples (200 cases:200 controls). Considering a variance inflation factor of 1.2 for adjustment of maternal factors and other covariates, the study will need to recruit 480 couples (240 cases:240 controls).

Previous studies have reported the use of the event per variable (EPV) ratio:the number of events (success pregnancy) divided by the number of regression coefficients in the model to determine the sample size of a multivariable model.^57^ An EPV of at least 10 has been recommended to avoid overfitting.^58^ We estimate 15 regression coefficients based on the independent male predicting factors of successful pregnancy in the logistic model. Following this guideline, we require 150 events. Given the observed pregnancy rate of 48% in the population,^59^ the total sample size calculated to achieve this number of events is approximately 313 (150/0.48). By accounting for a variance inflation rate of 1.2 for correlation between male and female factors, we will require 376 couples (188 cases:188 controls) in the study.

### Ethics and dissemination

The study has been approved by the Centralised Institutional Review Board of SingHealth (2024/2120), Singapore. The study will be performed in accordance with the ethical principles in the Declaration of Helsinki. Written informed consent will be obtained from all participants. Findings from this study will be disseminated via peer-reviewed publications, conference presentations and reports to the funding body. The data that support the findings of this study are available from the corresponding author on reasonable request.

## Discussion

In this study, we will comprehensively investigate multiple factors in males, including clinical, biological, behavioural, lifestyle, environmental and molecular characteristics, to understand their relationships with male reproductive health and successful pregnancy. State-of-the-art automated technology integrated with artificial intelligence algorithms will be used to measure sperm DNA fragmentation and assess biofluid oxidative status. This approach will provide crucial insights for reproductive healthcare, defining male fertility status and guiding fertility treatment decisions. Additionally, the assessment of sperm gene expression and DNA methylation will allow understanding of how male exposures affect fertility at a molecular level, aiding in identifying novel candidate biomarkers for predicting the chances of conception and potentially offspring health. These advancements represent a significant step towards implementing an entirely new class of precision medicine in male fertility care, enabling personalised management plans.

Furthermore, the study aligns with the POHaD paradigm, which recognises optimal paternal health as an essential component, along with maternal health, to create a favourable environment that has tremendous potential to influence the birth and growth of healthy children. These efforts will help mitigate the burden of the transgenerational cycle of chronic disease development within families and the wider population. Importantly, the findings of this study will raise awareness among healthcare agencies and the general public about the significant role of men (an essential but often neglected component) and the importance of optimising men’s health during preconception to improve reproductive health outcomes. By focusing on the father’s role in reproductive health outcomes through an evidence-based approach, there is a move to address the disproportionate burden placed on women and promote equity in healthcare and society, in addition to the growing evidence of the influence of sperm quality on reproductive outcomes.

Several limitations exist within this study. First, self-reported dietary and lifestyle information may be subjected to recall bias, potentially compromising the predictive accuracy of successful pregnancy. Individuals may provide biased estimates of self-assessed behaviour due to various factors, including misunderstanding or social desirability. While these types of bias are inherent to some extent, we endeavour to minimise them by using standardised and well-structured questionnaires and by avoiding long recall periods as much as possible. Second, given the study’s observational design, any causal link between risk factors and male subfertility remains inferential, with the potential for residual confounding. To address this limitation, we will use propensity score methods and conduct sensitivity analyses, including interaction testing and stratification by key confounders, to enhance the robustness of our findings. Additionally, our recruitment is exclusive to Singapore residents, potentially limiting the broader applicability of our results to other contexts. However, given the multiracial population of Chinese, Malay and Indian, the results obtained are likely generalisable to Asians.
