# Supplementary material for: Investigating male factors and their relationships with reproductive health outcomes: a case-control study protocol for Towards Optimal Fertility, Fathering, and Fatherhood studY (TOFFFY) in Singapore
Source: BMJ Open. 2025 Jan 15;15(1):e088143. doi: 10.1136/bmjopen-2024-088143 (PMC11751997; doi:10.1136/bmjopen-2024-088143)
Supplement: Supplementary file 1 [file bmjopen-2024-088143.reviewer_comments.pdf]

PEER REVIEW HISTORY

BMJ Open publishes all reviews undertaken for accepted manuscripts. Reviewers are asked to complete a checklist review form and are provided with free text boxes to elaborate on their assessment. These free text comments are reproduced below.

ARTICLE DETAILS

Title (Provisional)

Investigating male factors and their relationships with reproductive health outcomes: a case-control study protocol for Towards Optimal Fertility, Fathering, and Fatherhood studY (TOFFFY) in Singapore

Authors

Ku, Chee Wai; Pek, Jun Wei; Cheung, Yin Bun; Tharmalingam Durgahshree, Melissa D/O; Chan, Melinda; Lee, Yie Hou; Godfrey, Keith; Yap, Fabian; Chan, Jerry; Loy, See Ling

---

VERSION 1 - REVIEW

**Reviewer**	**1**
**Name**	**Gibreel, Ahmed**
**Affiliation**	**Mansoura University, Obstetrics and Gynecology Department**
**Date**	**24-May-2024**
**COI**	**None**

Well written protocol. However need to address the following: some researchers referred to 24 weeks of exposure rathar than 12 months to diagnose subfertility. Semen parameters may be affected above 40, DNA fragmentation assessment and managment may vary in terms of method of assessment and treatment.

**Reviewer**	**2**
**Name**	**Qin, Zuoshu**
**Affiliation**	**Tongji University, Department of Obstetrics and Gynecology**
**Date**	**26-Jul-2024**
**COI**	**I declare that I have no competing interests with the authors of this article.**

Thank you for submitting the study protocol titled "Investigating male factors and their relationships with reproductive health outcomes: a case-control study protocol for Towards Optimal Fertility, Fathering, and Fatherhood study (TOFFFY) in Singapore". I have thoroughly reviewed the protocol and provide the following feedback, which I hope will help improve the study design and methods.

Here are the main concerns:

1.Since this study is an observational design, it cannot determine the causal relationship between the observed factors and male infertility, and there may be some unmeasured or unknown confounding factors affecting the results. The method of balancing these confounding factors should be described in more detail in the article.

2.The analysis of DNA methylation is mentioned in the article, and it is hoped that the details of this analysis can be described more thoroughly.

VERSION 1 - AUTHOR RESPONSE

Reviewer 1
1. Well written protocol. However need to address the following: some researchers referred to 24 weeks of exposure rathar than 12 months to diagnose subfertility.

Response: Thank you for your comment. The American Society for Reproductive Medicine has defined infertility as the failure to achieve a successful pregnancy after 12 months of regular unprotected sexual intercourse (PMID: 34607703). Similarly, the National Institute for Health and Care Excellence (NICE) guidelines for Fertility Problems (PMID: 32134604) have also echoed the need to institute fertility assessments after 12 months of unprotected intercourse, because over 80% of couples in the general population will conceive within one year. We have included this definition, and the details of the selection of cases, as follows:

Line 80 – 81: Subfertility is defined as the failure to achieve conception after 12 months of regular, unprotected sexual intercourse.[5,6]

Line 198 – 199: Couples who are not able to conceive for at least 12 months of regular, unprotected intercourse.

2. Semen parameters may be affected above 40, DNA fragmentation assessment and managment may vary in terms of method of assessment and treatment.

Response: Thank you for the suggestion. While the associations between advanced paternal age, semen quality, and sperm DNA fragmentation are well-reported, there is no universally accepted definition of 'old age' in fathers. We have elaborated on this point in the 'Introduction'. Additionally, we have added detailed methods for assessing sperm DNA fragmentation in the 'Methods' section as follows:

Line 97 – 102: Advanced paternal age has been associated with subfertility, delayed embryo development, increased miscarriage risk and preterm birth, which can be attributable to poor DNA integrity and aberrant DNA methylation in sperm at older age.[13,14] However, the specific age at which reproductive potential begins to decline remains undefined.[13,15] Some studies

report changes starting around age 40;[16] others indicate pronounced declines in semen quality by age 50.[13,15]

Line 142 – 144: Single and double-stranded DNA breaks in sperm have different reproductive consequences.[36] Increased reactive oxygen species causes single stranded DNA breaks in sperm.

Line 146 – 148: Double stranded DNA breaks in sperm has been linked with suboptimal embryo development, poor implantation rates, and recurrent miscarriage in couples without a female factor.[37–39]

Line 261 – 263: Single and double stranded DNA breaks in sperm will be measured using the sperm chromatin dispersion assay (LensHooke® R10 Plus kit) and sperm DNA fragmentation releasing assay (LensHooke® R11 Plus kit) respectively.[53]

Reviewer 2

1. Since this study is an observational design, it cannot determine the causal relationship between the observed factors and male infertility, and there may be some unmeasured or unknown confounding factors affecting the results. The method of balancing these confounding factors should be described in more detail in the article.

Response: Thank you for the suggestion. We agree that addressing confounding is a critical aspect of observational studies. To address this, we have provided additional details in the 'Statistical Analysis Plan' and 'Limitations' sections, outlining the methods employed to minimize confounding, as follows:

Line 298 – 301: We will identify these potential confounders through a literature review and conceptual framework, guided by directed acyclic graphs. We will explore propensity score methods, such as inverse probability weighting, to ensure balanced distribution of confounders across case and control groups.

Line 344 – 346: To further ensure robustness, we will test interactions between male and female factors and stratify analyses by key confounders.

Line 413 – 415: To address this limitation, we will explore propensity score methods and conduct sensitivity analyses, including interaction testing and stratification by key confounders, to enhance the robustness of our findings.

2. The analysis of DNA methylation is mentioned in the article, and it is hoped that the details of this analysis can be described more thoroughly.

Response: We have now expanded the section describing the DNA methylation analysis in greater details, as follows:

Line 285 – 292: Genome-wide bisulfite sequencing (to profile global DNA methylation) for sperm from cases and controls will be performed as previously described.[57] Briefly, genomic DNA from sperm cells will be extracted and treated with bisulfite using the EZ DNA Methylation Lightening Kit (Zymo Research) in accordance with the manufacturer's protocol. The samples were further subjected to PCR amplification and sequencing using the Illumina

platform. Top differentially methylated genes will be selected based on their known functions in spermatogenesis, fertility and metabolic pathways such as insulin signaling, and further verified by targeted bisulfite sequencing using the remaining sperm samples from the cohort.

VERSION 2 - REVIEW

**Reviewer**	**1**
**Name**	**Gibreel, Ahmed**
**Affiliation Department**	**Mansoura University, Obstetrics and Gynecology**
**Date**	**06-Dec-2024**
**COI**	

This is accepted by myself now.
